# Comparison of Beiyi^®^ and its reference product Enantone^®^ as agonists of luteinizing hormone-releasing hormone against prostate cancer: a multicenter, randomized single-dose study

**DOI:** 10.3389/fphar.2026.1873100

**Published:** 2026-08-25

**Authors:** Yuyao Yi, Pengfei Shen, Tongsen Zheng, Ruigang Hou, Lianlian Fan, Yanxia Yu, Jian Li, Yu Fang, Chuan Hao, Pinghong You, Xiaoyu Lu, Xiongbing Lu, Qiang Wei, Ping Feng

**Affiliations:** 1 Clinical Trial Center, West China Hospital, Sichuan University, Chengdu, Sichuan, China; 2 Department of Urology, Institute of Urology, West China Hospital, Sichuan University, Chengdu, Sichuan, China; 3 Clinical Trial Center, Harbin Medical University Cancer Hospital, Harbin, Heilongjiang, China; 4 Clinical Trial Center, Second Hospital of Shanxi Medical University, Taiyuan, Shanxi, China; 5 Clinical Trial Center, Deyang People’s Hospital, Deyang, Sichuan, China; 6 Clinical Trial Center, Affiliated Suzhou Hospital of Nanjing Medical University, Suzhou Municipal Hospital, Suzhou, Jiangsu, China; 7 Clinical Trial Center, Second Affiliated Hospital of Nanchang University, Nanchang, Jiangxi, China; 8 Department of Urology, Institute of Urology, Second Hospital of Shanxi Medical University, Taiyuan, Shanxi, China; 9 Department of Urology, Institute of Urology, Deyang People’s Hospital, Deyang, Sichuan, China; 10 Department of Urology, Institute of Urology, Second Affiliated Hospital of Nanchang University, Nanchang, Jiangxi, China

**Keywords:** Beiyi^®^, bioequivalence (BE), Enantone^®^, leuprolide, pharmacokinetics, safety

## Abstract

**Background:**

Leuprolide acetate is a potent agonist of luteinizing hormone-releasing hormone that has been licensed to treat prostate cancer. The microsphere formulation of leuprolide acetate called Beiyi®, developed in China, has shown favorable pharmacokinetics, pharmacodynamics and safety similar to those of the reference drug Enantone® in healthy individuals. The present study examined whether the two formulations are bioequivalent in men with prostate cancer.

**Methods:**

This multicenter, open-label study randomized 120 Chinese men with prostate cancer 1:1 to receive a single subcutaneous injection of Beiyi® or Enantone® (3.75 mg). The two groups were compared in terms of pharmacokinetics and safety.

**Results:**

The ratio of the geometric means of the maximum serum concentration (C_max_) of Beiyi® and Enantone® was 98.34% (91.88%–105.25%); the ratio of the geometric means of the concentration-time curve from 7 to 28 days (AUC_7-28_) was 100.52% (88.17%–114.60%); and the ratio of the geometric means of the concentration-time curve from 0 to 28 days (AUC_0-28_) was 109.07% (98.54%–120.73%). The three 90% confidence intervals fell within the range of 80.00%–125.00%, indicating similar pharmacokinetics. The two drugs showed similar safety profiles, with no treatment-emergent adverse events leading to withdrawal or death.

**Conclusion:**

The two formulations of leuprolide acetate microspheres Beiyi® and Enantone® show similar pharmacokinetics and safety in Chinese men with prostate cancer. These results support the further clinical development of Beiyi® as a generic formulation of Enantone®.

**Trial Registration:**

CTR20222654 on http://www.chinadrugtrials.org.cn/index.html.

## 1 Introduction

Prostate cancer is a major health problem among men, and it is expected to become the most frequent type of cancer in American men in 2025 (Siegel et al., 2025), while it was the sixth most frequent cancer among men in China in 2022 (Han et al., 2024). A widespread treatment for advanced prostate cancer is hormone therapy designed to remove or antagonize the androgens that drive the growth and development of the prostate gland (Massard and Fizazi, 2011; Cornford et al., 2017; Hussain et al., 2013), which can involve surgical or chemical castration to prevent androgen synthesis and drugs that inhibit androgen activity or their transformation by 5α-reductase (Massard and Fizazi, 2011; Liede et al., 2016). Widely used for chemical castration are agonists of luteinizing hormone-releasing hormone (LHRH)—also known as gonadotropin-releasing hormone (GnRH)—such as leuprolide, goserelin, and buserelin (Massard and Fizazi, 2011; Hussain et al., 2013; Perlmutter and Lepor, 2007; Uemura et al., 2020).

One of the most effective LHRH agonists is leuprolide acetate, which stimulates luteinizing hormone releasing about 100-fold better and inhibits the pituitary-gonadal system more strongly than natural LHRH (Plosker and Brogden, 1994; Wilson et al., 2007). It is also strongly resistant to proteolytic digestion, it strongly binds LHRH receptors, and it shows good tolerability (Abouelfadel and Crawford, 2008; Özyiğit, 2020).

Various formulations of leuprolide acetate designed to release the drug slowly have been developed, such as Lupron depot®, Eligard®, and Enantone® (Shore et al., 2019; de Freitas and Soares, 2020; Bruneu-Avierinos et al., 2011; Suzuki et al., 2015). Enantone®, manufactured collaboratively by US and Japanese companies and approved for the clinic in 1989 by the Food and Drug Administration in the US and in 2003 by the State Food and Drug Administration in China, is a formulation of leuprolide acetate in microspheres based on lactic acid polymers (Bruneu-Avierinos et al., 2011; Suzuki et al., 2015). In 2009, the State Food and Drug Administration approved a second injectable microsphere formulation of leuprolide acetate called Beiyi®, manufactured by a Chinese company. In healthy individuals, Beiyi® has shown pharmacokinetics, pharmacodynamics, tolerability and safety similar to those of Enantone®, while inducing potentially higher castration rates and longer castration times than Enantone® (Hu et al., 2022).

In China, there have been two previous clinical trials of bioequivalence of leuprolide acetate microspheres in healthy participants, but none in patients with prostate cancer yet. As a further step towards determining whether the two LHRH agonist formulations Beiyi® and Enantone® can be considered equivalent, the present trial compared their pharmacokinetics and safety in men with prostate cancer.

## 2 Methods

### 2.1 Ethics approval

This clinical trial was approved by the Ethics Committee of our institutions and was performed in accordance with the Helsinki Declaration and with Good Clinical Practice guidelines of the International Council for Harmonisation of Technical Requirements for Pharmaceuticals for Human Use. Before enrollment, patients were informed in writing about the purpose, procedures, risks as well as potential side effects and drug interactions related to participation in the study. All patients had to sign written informed consent in order to be enrolled.

### 2.2 Study population

To be enrolled in the study, subjects had to be men with prostate cancer 18–75 years old with a body weight no less than 50 kg and a body mass index of 19.0–30 kg/m^2^. Subjects could not show clinically significant abnormalities during physical examination at screening, or during any of the following tests at screening: 12-lead electrocardiography, vital signs, complete blood count and blood biochemistry, urinalysis, coagulation function, and glycated hemoglobin level.

Patients were excluded from consideration if they had a history of severe cardiovascular and cerebrovascular diseases, adrenalectomy, pituitaryectomy, pituitary disorder; history of other malignancies within the previous 5 years, unless it was basal cell carcinoma or squamous cell carcinoma of the skin that have been surgically removed; or history of androgen deprivation therapy for prostate cancer, unless it was leuprolide acetate microsphere injection. Patients were also excluded if they had an infection at screening.

### 2.3 Study design and sample size

This multicenter phase I bioequivalence study relied on a randomized, open-label, single-dose, parallel-group design. The participants were Chinese men with prostate cancer who were already receiving subcutaneous injections of leuprolide acetate microspheres at 3.75 mg, or who had never received such injections or any other androgen-deprivation therapy.

Our plan was to enroll a sample large enough to detect ratios of geometric means for the pharmacokinetic parameters of area under the concentration-time curve (AUC) and maximum serum concentration (C_max_) for the Beiyi® and Enantone® groups between 0.95 and 1.05 with a unilateral α = 0.05 and power of 1-β = 0.8. Given the highest inter-individual coefficient of variation for AUC around 34% in a comparison of Beiyi® and Enantone® in healthy men (Hu et al., 2022), the software PASS 11.0 (NCSS LLC., Kaysville, U.T., United States) indicated a minimal sample of 96 patients. We increased this to 120 to compensate for up to 20% dropout.

### 2.4 Group allocation and study treatment

Eligible patients were randomized in a 1:1 ratio to receive either investigational product (Beiyi®, batch 220201, Shanghai Livzon Pharmaceutical, Shanghai, China) or reference product (Enantone®, batch 504860, Takeda Pharmaceutical, Yamaguchi, Japan) using a stratified block randomization method. The stratification factors were study center and treatment regimen (initial treatment vs. maintenance treatment). Patients in the maintenance treatment group were required to undergo a 28-day run-in period before entering the study phase; this involved a single subcutaneous injection of 3.75 mg of Beiyi® on Day −28. Patients in the initial treatment group entered the study phase directly. On Day 0, subjects in the study phase received a subcutaneous injection of either the Beiyi® or the reference product Enantone® according to their randomization assignment. Blood samples and relevant assessments were collected as specified in the protocol, and subjects were discharged from the study after completion of all blood sampling and assessments on Day 28.

### 2.5 Pharmacokinetics

Plasma leuprorelin concentrations were quantified via LC-MS/MS. The lower limit of quantification (LLOQ) was 0.0100 ng/mL, with linearity established from 0.0100 to 10.00 ng/mL (R^2^ = 0.9954–0.9996). Across 21 sample batches, inter-batch precision and accuracy within the linear range ranged from 1.56% to 6.33% and 99.41%–100.74%, respectively.

Based on a time to maximum concentration (T_max_) of 1–4 h for leuprolide in injectable leuprolide acetate microspheres (Takeda Pharmaceutical Company Limited, 2024), blood was sampled for pharmacokinetic analysis at the following time points (h): 0 (within 1 h before dosing), 0.5, 1.0, 1.5, 2.0, 2.5, 3.0, 6.0, 12.0, 24 (day 1), 48.0 (day 2), 72.0 (day 3), 168.0 (day 7), 336.0 (day 14), 504.0 (day 21), and 672.0 (day 28).

Blood was analyzed for AUC from day 7 to day 28 (AUC_7-28_), AUC from baseline until day 28 (AUC_0-28_), C_max_, T_max_, terminal rate constant (λz), terminal half-life (t_½_), systemic clearance (CL/F), and apparent volume of distribution (V_d_/F). These parameters were determined using a non-compartment model in Phoenix WinNonlin 8.3 (Certara United States, Inc., Princeton, NJ).

### 2.6 Safety

At day 28, patients underwent a full safety assessment involving 12-lead electrocardiography, vital signs, physical examination, complete blood count and blood biochemistry, urinalysis, coagulation function, and glycated hemoglobin level. Adverse events during the 28-day trial were coded according to MedDRA 26.0 (The International Council for Harmonisation of Technical Requirements for Pharmaceuticals for Human Use, ICH) and graded by severity according to CTCAE 5.0 (National Cancer Institute, NCI).

### 2.7 Statistical analysis

Data were analyzed statistically using SAS® 9.4 (SAS Institute, Cary, NC, United States). Differences in log-transformed data were assessed for significance using analysis of variance. T_max_ were assessed using the Wilcoxon rank sum test. The two drugs were considered bioequivalent if the 90% confidence intervals for the ratios of the geometric means of AUC_7-28_, AUC_0-28_ and C_max_ fell within the range 80.00%–125.00% (Center for Drug Evaluation of National Medical Products Administration, 2016).

## 3 Results

**FIGURE 1:**
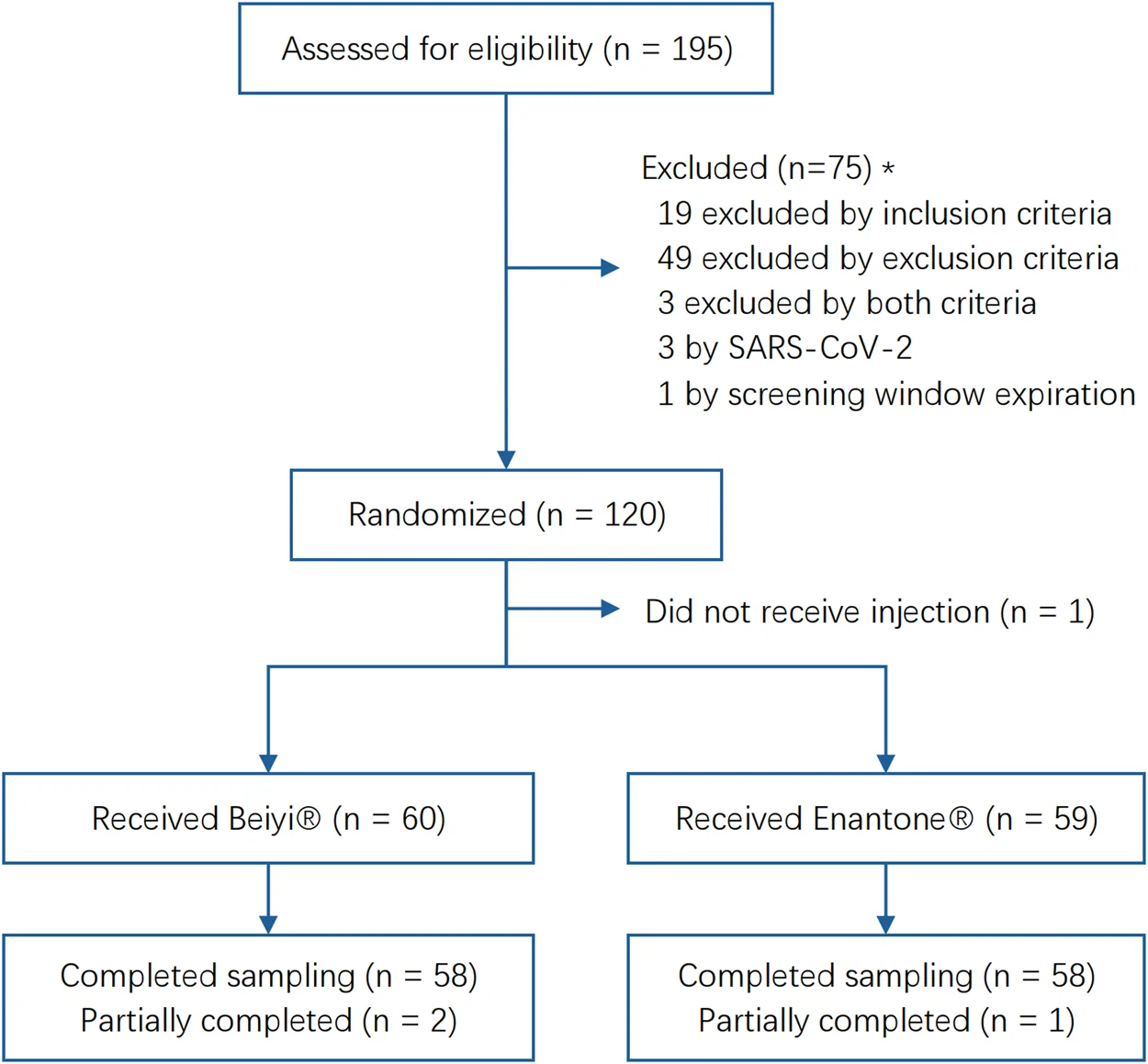
Flowchart of enrollment, treatment and analysis of patients. * exact reasons are listed in Supplementary Table S4.

Of the 195 patients at 6 medical centers in China who were screened, 120 were enrolled (Figure 1). They were randomized 1:1 into the Beiyi® and Enantone® study arms, After randomization, one patient withdrew for personal reasons. The remaining 119 patients received the single injection of assigned study drug, and were included in final analyses of pharmacokinetics and safety. Among the participants who completed the trial, all but four were Han Chinese, age ranged from 54 to 75 years, height from 147.0 to 180.0 cm, weight from 50.6 to 92.0 kg, and body mass index from 19.2 to 29.8 kg/m^2^ (Table 1). The two study arms did not present any notable differences at baseline.

**TABLE 1:** Characteristics of men with prostate cancer at baseline.

Characteristic	Beiyi® group	Enantone® group
n	60	59
Prior GnRH analog therapy	58	58
Age, yr	66.8 ± 5.8	66.6 ± 5.4
Weight, kg	68.1 ± 9.5	69.3 ± 8.2
Height, cm	164.5 ± 7.3	165.5 ± 5.1
Body mass index, kg/m^2^	25.1 ± 2.5	25.3 ± 2.3
Male	60 (100)	59 (100)
Asian	60 (100)	59 (100)
Ethnicity	​	​
Han	58 (96.7)	57 (96.6)
Man	2 (3.3)	0
Qiang	0	1 (1.7)
Hui	0	1 (1.7)

Values are mean ± SD, or n (%).

### 3.1 Pharmacokinetics

**FIGURE 2:**
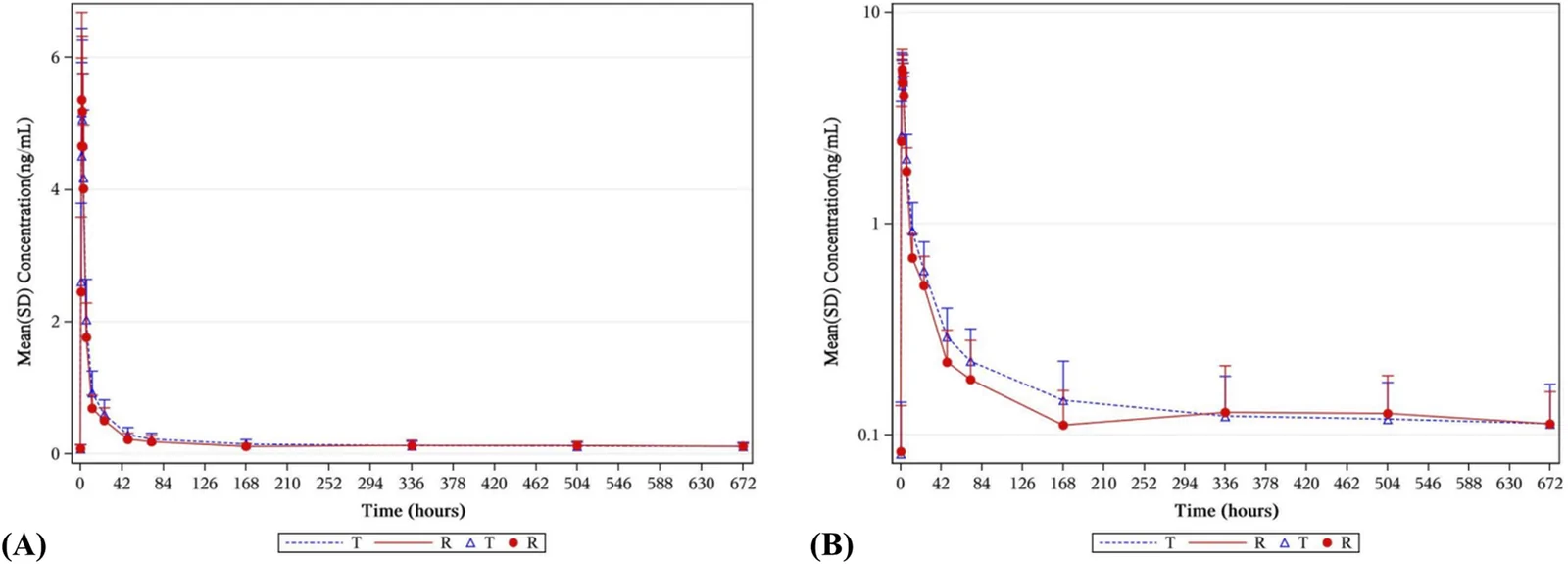
Concentration of leuprolide in serum as a function of time after a single subcutaneous dose of 3.75 mg. Values are mean ± SD. Data were plotted on a **(A)** linear or **(B)** semi-logarithmic y-axis.

The average serum concentration-time curves after a single subcutaneous injection of either Beiyi® or Enantone® were quite similar (Figure 2), as were the various pharmacokinetic parameters that we examined (Table 2). The ratios of the geometric means for the two drugs were 98.34% (91.88%–105.25%) for C_max_, 100.52% (88.17%–114.60%) for AUC_7-28_, and 109.07% (98.54%–120.73%) for AUC_0-28_ (Table 3). Median T_max_ was not significantly different between Beiyi® (1.50 h, range 0.98–2.52 h) and Enantone® (1.50 h, range 0.5–2.5 h; Supplementary Table S1). Similar pharmacokinetic results were obtained whether or not we included one patient in the Beiyi® arm whose serum concentration of leuprolide at baseline (0.2353 ng/mL) exceeded 5% of C_max_ after dosing (4.397 ng/mL) (Supplementary Tables S2-S3). Because the 90% confidence intervals for the main pharmacokinetic parameters in this trial fell within the predefined range of 80.00%–125.00%.

**TABLE 2:** Comparison of pharmacokinetic parameters between Beiyi^®^ and Enantone^®^ after a single subcutaneous injection of 3.75 mg.

Parameter	Beiyi® group (n = 60)	Enantone® group (n = 59)
T_max_, h	1.50 (0.98, 3)	1.50 (0.5, 2.5)
C_max_, ng/mL	5.48 ± 1.18 (21.56)	5.59 ± 1.23 (21.91)
AUC_7-28_, h • ng/mL	62.26 ± 27.51 (44.18)	60.38 ± 28.56 (47.30)
AUC_0-28_, h • ng/mL	136.40 ± 44.56 (32.67)	123.04 ± 44.30 (36.00)
λz, 1/h	0.0027 ± 0.0017 (64.51)	0.0056 ± 0.0153 (271.87)
t_1/2_, h	485.19 ± 601.05 (123.88)	416.74 ± 1208.17 (289.91)
CL/F, L/h	21.62 ± 8.77 (40.55)	27.16 ± 14.20 (52.29)
V_d_/F, L	11,833.61 ± 8156.92 (68.93)	8709.74 ± 5179.84 (59.47)

Values are median (interquartile range) or mean ± SD (coefficient of variation, %).

**TABLE 3:** Assessment of bioequivalence between Beiyi^®^ and Enantone^®^ after a single subcutaneous injection of 3.75 mg.

Parameter	Group	n*	GLSM	Ratio of Beiyi® to Enantone®, %		CVi%	PT%
				Value	90%CI		
C_max_, ng/mL	Beiyi®	59	5.37	98.3	91.9–105.3	22.52	99.96
​	Enantone®	59	5.46	​	​	​	​
AUC_7-28_, h • ng/mL	Beiyi®	57	56.47	100.5	88.2–114.6	44.36	75.34
​	Enantone®	58	56.18	​	​	​	​
AUC_0-28_, h • ng/mL	Beiyi®	57	128.66	109.1	98.5–120.7	33.73	71.47
​	Enantone®	58	117.95	​	​	​	​

CI, confidence interval; CVi, coefficient of intra-individual variation; GLSM, generalized least squares mean; PT, power of test.

* One subject in the Beiyi® group had a serum concentration before dosing more than 5% maximum and was therefore excluded from all calculations. Another two subject in the Beiyi® group and one subjects in the Enantone® group were excluded from AUC, calculations because blood was not sampled from them at all predefined time points.

### 3.2 Safety

Among the 119 patients who received the study drug, 53 experienced 95 adverse events during the 28-day observation period. Four of those adverse events preceded dosing with study drug, leaving 91 treatment-emergent adverse events affecting 51 patients, corresponding to an overall incidence of 42.9% (51/119) and incidence of 45.0% (27/60) in the Beiyi® group and 40.7% (24/59) in the Enantone® group (Table 4). The only serious treatment-emergent events were one case of vertigo and one case of acute exacerbation of chronic bronchitis, both in the Beiyi® group. None of the treatment-emergent adverse events led to patient withdrawal, and none of the patients in the study died.

**TABLE 4:** Comparison of adverse events in patients between Beiyi^®^ and Enantone^®^ after a single subcutaneous injection of 3.75 mg.

Adverse event	Beiyi® group (n = 60)	Enantone® group (n = 59)	All patients (N = 119)
All events	28 (46.7), 49	25 (42.4), 46	53 (44.5), 95
Treatment-emergent events			
All	27 (45.0), 48	24 (40.7), 43	51 (42.9), 91
Serious	2 (3.3), 2	0 (0.0), 0	2 (1.7), 2
Led to withdrawal	0 (0.0), 0	0 (0.0), 0	0 (0.0), 0
Treatment-related events			
All	21 (35.0), 31	19 (32.2), 27	40 (33.6), 58
Serious	0 (0.0), 0	0 (0.0), 0	0 (0.0), 0
Led to death	0 (0.0), 0	0 (0.0), 0	0 (0.0), 0

Values indicate “n (%) of patients, n of events”.

Of the 48 treatment-emergent adverse events in the Beiyi® group, 25 were grade I severity, 19 were grade II, 3 were grade III, and 1 were grade IV. Of the 43 treatment-emergent adverse events in the Enantone® group, 34 were grade I severity and 9 were grade II. In both study arms, the most frequent types of treatment-emergent adverse events were metabolic or nutritional disorders, abnormal test results, and infectious diseases; and the most frequently occurring terms related to COVID-19, hypertriglyceridemia and hypercholesterolemia (Table 5).

**TABLE 5:** Comparison of treatment-emergent adverse events between the Beiyi^®^ and Enantone^®^ after a single subcutaneous injection of 3.75 mg.

Event	Beiyi® group	Enantone® group	All patients
All events	27 (45.0), 48	24 (40.7), 43	51 (42.9), 91
Metabolic and nutritional disorders	13 (21.7), 16	6 (10.2), 8	19 (16.0), 24
Hypertriglyceridemia	5 (8.3), 5	3 (5.1), 3	8 (6.7), 8
Hypercholesterolemia	4 (6.7), 4	3 (5.1), 3	7 (5.9), 7
Hyperlipidemia	2 (3.3), 3	1 (1.7), 1	3 (2.5), 4
Hyperglycemia	2 (3.3), 2	1 (1.7), 1	3 (2.5), 3
Diabetes mellitus	1 (1.7), 1	0 (0), 0	1 (0.8), 1
Hypokalemia	1 (1.7), 1	0 (0), 0	1 (0.8), 1
Clinical investigations	7 (11.6), 8	8 (13.6), 11	15 (12.6), 19
Elevated glycated hemoglobin	3 (5.0), 3	4 (6.8), 4	7 (5.9), 7
Hypertension	2 (3.3), 3	0 (0), 0	2 (1.7), 3
Elevated blood bilirubin	1 (1.7), 1	1 (1.7), 1	2 (1.7), 2
Prolonged corrected QT interval on electrocardiography	1 (1.7), 1	1 (1.7), 1	2 (1.7), 2
Elevated CPK	0 (0), 0	1 (1.7), 1	1 (0.8), 1
Abnormal T wave on electrocardiography	0 (0), 0	1 (1.7), 1	1 (0.8), 1
Elevated aspartate aminotransferase	0 (0), 0	1 (1.7), 1	1 (0.8), 1
Elevated alanine aminotransferase	0 (0), 0	1 (1.7), 1	1 (0.8), 1
Elevated GGT	0 (0), 0	1 (1.7), 1	1 (0.8), 1
Infections and infestations	7 (11.7), 7	9 (15.3), 9	16 (13.4), 16
COVID-19	6 (10.0), 6	8 (13.6), 8	14 (11.8), 14
Bronchial infection	1 (1.7), 1^a^	0 (0), 0	1 (0.8), 1
Urinary tract infection	0 (0), 0	1 (1.7), 1	1 (0.8), 1
Vascular disorders	5 (8.3), 6	3 (5.1), 4	8 (6.7), 10
Hypertension	4 (6.7), 5^b^	0 (0), 0	4 (3.4), 5
Hot flashes	0 (0), 0	2 (3.4), 2	2 (1.7), 2
Hypotension	1 (1.7), 1	1 (1.7), 1	2 (1.7), 2
Hematoma	0 (0), 0	1 (1.7), 1	1 (0.8), 1
Blood or lymphatic disorders	1 (1.7), 1	2 (3.4), 2	3 (2.5), 3
Anemia	1 (1.7), 1	2 (3.4), 2	3 (2.5), 3
Injury, poisoning or procedural complications	0 (0), 0	1 (1.7), 3	1 (0.8), 3
Bruising	0 (0), 0	1 (1.7), 2	1 (0.8), 2
Thoracic trauma	0 (0), 0	1 (1.7), 1	1 (0.8), 1
Musculoskeletal or connective tissue disorders	2 (3.3), 2	1 (1.7), 1	3 (2.5), 3
Facet joint osteoarthritis	1 (1.7), 1	0 (0), 0	1 (0.8), 1
Back pain	0 (0), 0	1 (1.7), 1	1 (0.8), 1
Pain in extremity	1 (1.7), 1	0 (0), 0	1 (0.8), 1
Gastrointestinal disorders	1 (1.7), 1	1 (1.7), 1	2 (1.7), 2
Tongue pain	1 (1.7), 1	0 (0), 0	1 (0.8), 1
Abdominal pain	0 (0), 0	1 (1.7), 1	1 (0.8), 1
Hepatobiliary disorders	1 (1.7), 1	1 (1.7), 1	2 (1.7), 2
Gallstone disease	0 (0), 0	1 (1.7), 1	1 (0.8), 1
Abnormal liver tests	1 (1.7), 1	0 (0), 0	1 (0.8), 1
Ear or labyrinth disorders	1 (1.7), 2	0 (0), 0	1 (0.8), 2
Vertigo	1 (1.7), 2^c^	0 (0), 0	1 (0.8), 2
Respiratory, thoracic or mediastinal disorders	2 (3.3), 2	0 (0), 0	2 (1.7), 2
Chronic bronchitis	1 (1.7), 1	0 (0), 0	1 (0.8), 1
Cough	1 (1.7), 1	0 (0), 0	1 (0.8), 1
General disorders or administration site conditions	0 (0), 0	2 (3.4), 2	2 (1.7), 2
Injection site reaction	0 (0), 0	1 (1.7), 1	1 (0.8), 1
Fever	0 (0), 0	1 (1.7), 1	1 (0.8), 1
Psychiatric disorders	0 (0), 0	1 (1.7), 1	1 (0.8), 1
Insomnia	0 (0), 0	1 (1.7), 1	1 (0.8), 1
Cardiac disorders	1 (1.7), 1	0 (0), 0	1 (0.8), 1
Myocardial ischemia	1 (1.7), 1	0 (0), 0	1 (0.8), 1
Nervous system disorders	1 (1.7), 1	0 (0), 0	1 (0.8), 1
Cerebrovascular ischemia	1 (1.7), 1	0 (0), 0	1 (0.8), 1

Values indicate “n (%) of patients, n of events”. GGT, gamma-glutamyl transferase.

^a^ graded IV.

^b^ 2 of five graded III.

^c^ graded III.

## 4 Discussion

Since the bioequivalence has already been shown in healthy men (Hu et al., 2022), the study justifies further exploration of Beiyi® as a generic formulation. In this sense, Beiyi® may complement another Chinese-produced generic microsphere formulation that has shown bioequivalence to Enantone® in healthy men (Zhou et al., 2021). Both generics have shown the ability to inhibit testosterone production and provide chemical castration similar or superior to Enantone® in healthy individuals.

Since our study did not identify any statistically significant differences in the pharmacokinetic parameters between Beiyi® and Enantone®. Indeed, our trial provides the first evidence that a generic formulation of leuprolide acetate microspheres is bioequivalent to Enantone® in men with prostate cancer in China. The observed 9% higher AUC_0–28_ in the Beiyi® group falls well within the bioequivalence range (80.00%–125.00%), indicating comparable systemic exposure. This difference likely stems from formulation-specific characteristics rather than baseline effects. For long-acting depots, variations in inactive ingredients and release kinetics can alter exposure profiles. However, since our study was conducted as a single-dose trial, future studies should investigate the effects of multiple doses on this parameter.

Although the lack of pharmacodynamic parameters (such as testosterone and luteinizing hormone) represents a limitation, pharmacokinetic parameters remain the primary standard for establishing bioequivalence; when the drug can be reliably quantified, pharmacodynamic parameters are not required (Kruse et al., 2025; Paixao et al., 2024). Furthermore, pharmacodynamic parameters are indirect indicators of drug activity, influenced by multiple factors including complex physiological feedback loops, baseline variability, and significant lag effects in hormonal responses. Given that only a single dose was administered in this study, the above-mentioned effects were more pronounced. The bioequivalence assessment method employed in this study is scientifically rigorous and reliable, sufficient to demonstrate the equivalence of the two leuprorelin formulations.

Following administration of Beiyi®, we observed three cases of Grade III adverse events and one case of Grade IV adverse event. One of the Grade III adverse events was vertigo. Vertigo was a subjective symptom reported by the patient, who had a long history of administering 3.75 mg of leuprorelin acetate microspheres for injection without experiencing vertigo-related adverse reactions. Literature reports (Chen et al., 2020) indicate no adverse reactions associated with vestibular dysfunction due to leuprorelin; therefore, this severe adverse event (grade III) is considered likely unrelated to Beiyi®. Grade IV adverse event was bronchial infection. The patient with acute exacerbation of chronic bronchitis had underlying medical conditions, and this episode represented an acute worsening. Leuprorelin does not have immunosuppressive effects; on the contrary, LHRH-induced androgen deprivation promotes thymus regeneration and enhances thymic output (Zhang et al., 2026). Additionally, leuprorelin improves T-cell immunity by inhibiting gonadal hormone production and restoring thymic activity (Gao et al., 2024), thus it would not predispose patients to bronchial infection. Additionally, this Grade IV serious adverse event manifested approximately 1 month after dosing. Consequently, we concluded that this event was definitely unrelated to Beiyi®. The other two grade III adverse events were hypertension, which are not considered serious adverse events; although we suspected a potential association with Beiyi®, both events subsequently resolved. We also observed 14 enrolled patients contracted COVID-19 during the study. Considering that the study was conducted amid the COVID-19 pandemic in China, these infections were unlikely to be related to either Beiyi® or Enantone®.

The types of treatment-emergent adverse events in our trial are consistent with the adverse events that have been associated with leuprolide in prostate cancer treatment (Plosker and Brogden, 1994; Shore et al., 2019; de Freitas and Soares, 2020; Bruneu-Avierinos et al., 2011; Suzuki et al., 2015) and that are listed in the package insert for Enantone® (Takeda Pharmaceutical Company Limited, 2024). Adverse events frequently reported with leuprolide acetate are hot flashes, hypertension and local pain at the injection site (Takeda Pharmaceutical Company Limited, 2024; Sountoulides and Rountos, 2013; Geiges et al., 2013), which occurred in only a few patients in our trial, which may reflect those participants received only a single dose. While our trial provides preliminary evidence of similar safety between Beiyi® and Enantone®, future trials should examine adverse events after multiple dosing and longer follow-up in order to detect, for example, increased risk of cardiovascular disease (Freedland and Abrahamsson, 2021).

Above all, our trial suggests that the Chinese-produced Beiyi® formulation of leuprolide acetate microspheres offers similar pharmacokinetics, tolerability and safety as the well-established and widely used Enantone® formulation in men with prostate cancer.

## 5 Conclusion

The generic formulation of leuprolide acetate microspheres Beiyi® shows similar pharmacokinetics and safety as the reference formulation Enantone® after a single subcutaneous dose of 3.75 mg in Chinese men with prostate cancer. These findings justify further development of Beiyi® as a generic formulation of Enantone®.

## Data Availability

The original contributions presented in the study are included in the article/Supplementary Material, further inquiries can be directed to the corresponding authors.
