# Supplementary material for: Comparison of Beiyi® and its reference product Enantone® as agonists of luteinizing hormone-releasing hormone against prostate cancer: a multicenter, randomized single-dose study
Source: Front Pharmacol. 2026 Aug 25;17:1873100. doi: 10.3389/fphar.2026.1873100 (PMC13550989; doi:10.3389/fphar.2026.1873100)
Supplement: Supplementary file 1 [file Supplementaryfile1.docx]

Supplementary Material

# 1 Supplementary Tables

Supplementary Table 1. Comparison of Tmax (in hours) between between Beiyi® and Enantone® after a single subcutaneous injection of 3.75 mg

| **Parameter** | **Beiyi® group** | **Enantone® group** | **Wilcoxon rank sum test value** | **P value** |
| --- | --- | --- | --- | --- |
| n | 59* | 59 | 1.1813 | 0.2375 |
| Mean ± SD | 1.70 ± 0.46 | 1.61 ± 0.43 |  |  |
| Median | 1.50 | 1.50 |  |  |
| Range | 0.98-2.52 | 0.5-2.5 |  |  |
| First-third quartiles | 1.50-2.00 | 1.50-1.98 |  |  |

* One subject in the Beiyi® group had a serum concentration of leuprolide more than 5% the maximum serum concentration after dosing and was therefore excluded from these calculations.

Supplementary Table 2. Sensitivity analysis of pharmacokinetics

| **ParametWer** | **Group** | **N** | **GLSM** | **T/R%** | **90%CI (%)** | **CVi%** | **PT%** |
| --- | --- | --- | --- | --- | --- | --- | --- |
| C_max_, ng/mL | T | 60 | 5.35 | 98.0 | 91.6-104.9 | 22.50 | 99.95 |
|  | R | 59 | 5.46 |  |  |  |  |
| AUC_7-28_, h×ng/mL | T | 58 | 56.59 | 100.7 | 88.5-114.7 | 44.16 | 76.20 |
|  | R | 58 | 56.18 |  |  |  |  |
| AUC_0-28_, h×ng/mL | T | 58 | 128.98 | 109.4 | 98.9-120.9 | 33.60 | 70.68 |
|  | R | 58 | 117.95 |  |  |  |  |

GLSM, generalized least squares mean; T group, Beiyi® group. R group, Enantone® group. T/R, Test/reference ratio. PT, power of test. CVi, Intra-individual variation.

One subject in test group and two subjects in reference group did not complete all sampling. One subject in test group with a serum higher than 5% Cmax before administration is included.

Supplementary Table 3. Wilcoxon rank sum test of Tmax (in hours) (sensitivity analysis)

|  | **Beiyi® group** | **Enantone® group** | **Statistics** | **P value** |
| --- | --- | --- | --- | --- |
| N (Missing) | 60(0) | 59(0) | -1.3277 | 0.1843 |
| Mean±SD | 1.72±0.48 | 1.61±0.43 |  |  |
| Median | 1.50 | 1.50 |  |  |
| Min～Max | 0.98～3 | 0.5～2.5 |  |  |
| Q1～Q3 | 1.50～2.00 | 1.50～1.98 |  |  |

One subject in test group with a serum higher than 5% Cmax before administration is included.

Supplementary Table 4. The reasons for exclusion and number of candidates in eligibility assessment

| **Reason** | **Excluded** |
| --- | --- |
| abnormal blood cell/liver/kidney test | 13 |
| severe cardiovascular or cerebrovascular diseases | 12 |
| Researcher's judgement | 9 |
| HBV/HCV/HIV/TP infection | 8 |
| HbA1c＞8% | 7 |
| difficulty in communication | 5 |
| BMI < 19 or >30 kg/m^2 | 4 |
| recently exposed to drug which prolong QT interval | 4 |
| COVID-19 | 3 |
| age < 18 or > 75 | 2 |
| substance abuse | 2 |
| have androgen deprived | 1 |
| participate in another trial | 1 |
| allergic to more than 2 substances | 1 |
| impaired micturition reflex | 1 |
| screening window expiration | 1 |
| need additional therapy | 1 |

If one subject is excluded by several reasons, the first reason in the inclusion and exclusion criteria is selected.
